# DHPSFU: a Fiji plugin for fast and accurate double helix-PSF 3D single-molecule localisation microscopy

**DOI:** 10.1038/s41598-025-15623-3

**Published:** 2025-08-20

**Authors:** Ziwei Zhang, Aleksandra Ochirova, Siqi Liu, Alex D. Herbert, Yunzhao Wu, Wayne Boucher, Steven F. Lee, Ernest D. Laue, David Klenerman, Aleks Ponjavic

**Affiliations:** 1 Yusuf Hamied Department of Chemistry, University of Cambridge, Cambridge, UK; 2 Department of Biochemistry, University of Cambridge, Cambridge, UK; 3 School of Physics and Astronomy, University of St. Andrews, St Andrews, UK; 4 Chromosome Dynamics and Stability Group, Genome Damage and Stability Centre, University of Sussex, Brighton, UK; 5 Wellcome-MRC Cambridge Stem Cell Institute, Jeffrey Cheah Biomedical Centre, Cambridge, UK; 6 School of Physics and Astronomy, University of Leeds, Leeds, UK; 7 School of Food Science and Nutrition, University of Leeds, Leeds, UK; 8 Department of Biochemistry and Molecular Cell Biology, Shanghai Key Laboratory for Tumor Microenvironment and Inflammation, Key Laboratory of Cell Differentiation and Apoptosis of Chinese Ministry of Education, Shanghai Jiao Tong University School of Medicine, Shanghai, China

**Keywords:** Nanoscale biophysics, Single-molecule biophysics, Super-resolution microscopy

## Abstract

The double-helix point-spread function (DH-PSF) is one of the most used PSFs for large depth-of-field 3D single-molecule localisation microscopy. Due to its popularity, many algorithms have been developed to analyse experimental DH-PSF data, either based on dedicated DH-PSF fitting or on generalised PSF fitting, typically using cubic splines. We show here that the most popular implementations of both these approaches have limitations in terms of localisation performance, processing speed or user-friendliness. To overcome some of these limitations, we have developed a new analytical approach for DH-PSF fitting based on unmixing (DHPSFU) of fitted localisation data using distance pairing. We compare DHPSFU with two popular algorithms, SMAP and EasyDHPSF, using realistic simulated datasets based on experimental data, to show that our algorithm achieves the highest Jaccard index (DHPSFU: 0.98; SMAP: 0.91; EasyDHPSF: 0.85) and fastest CPU-based processing speed (DHPSFU: 6,800 locs/s; SMAP: 2,500 locs/s; EasyDHPSF: 63 locs/s). We also show that our algorithm achieves the best resolution when imaging the cellular plasma membrane of Jurkat T cells (DHPSFU: 140 nm, EasyDHPSF: 162 nm, SMAP: 165 nm). We have incorporated DHPSFU as a Fiji plugin and provide Matlab and Python scripts for user customisation.

**Supplementary Information:**

The online version contains supplementary material available at 10.1038/s41598-025-15623-3.

## Introduction

Single molecule localisation microscopy (SMLM) techniques enable visualisation of subcellular structures, interactions and dynamics at the molecular scale, beyond the diffraction limit^1^. SMLM has been widely used in both biological^2^ and non-biological^3^ applications due to its high spatial resolution, typically around 20 to 50 nm, and its straightforward implementation in conventional wide-field microscopes. SMLM relies on detecting the stochastic blinking of individual fluorophores, whose positions can be accurately determined from a series of diffraction-limited images to build up a composite super-resolution image reconstruction.

SMLM has been extended to three dimensions using point-spread function (PSF) engineering methods, achieving sub-50-nm localisation precision in both the lateral and axial dimensions^4^. These methods break the axial symmetry of the PSF through the introduction of specific optical elements that modulate the emission based on axial depth. The axial range varies across different techniques, with common methods including astigmatism (~ 1 μm)^5^, biplane (~ 1 μm)^6^, double helix-PSF (DH-PSF) (3–4 μm)^7^, multi-focus microscopy (~ 4 μm)^8^, light-field microscopy (LFM; ~8 μm)^9^, and tetrapod-PSF (6–20 μm)^10^. Among these methods, DH-PSF microscopy stands out for its simplicity, excellent axial resolution, reasonable axial range of 3–4 μm and relatively compact PSF shape^11^. In DH-PSF microscopy a bespoke phase mask is placed in the emission path. As a result, the lateral cross-section of the fluorophore’s PSF is split into two lobes whose positions rotate according to the axial depth, forming a double helix (DH). DH-PSF microscopy provides a localisation precision of approximately 10 nm laterally and 30 nm axially throughout the entire 3–4 μm axial range^12^, and has been employed in various SMLM imaging and single-molecule tracking applications^13–16^.

Current algorithms for analysing DH-PSF image data typically proceed in two steps: (1) detection of localisations and their x- and y-coordinates, and (2) determination of the z-coordinate based on the PSF shape. Approaches for localisation detection include template matching by cross-correlation (e.g. EasyDHPSF^17,18^) or peak detection in a mean-filtered image (e.g. SMAP^19^). Determining the axial position usually involves calculating the angle between the two DH-PSF lobes for localisation and comparing this with the known angular z-dependency obtained from a stack of calibration images. The calibration models vary in their complexity and underlying assumptions, ranging from a simple description of the DH-PSF as a rotating pair of 2D Gaussian peaks as for EasyDHPSF to a more flexible approach using cubic splines for SMAP. While these algorithms have been found to achieve over 95% detection precision^20^, this is not seen with all datasets and they have limitations in terms of user-friendliness – e.g. requiring separate installation, knowledge of programming, or limited processing speed, taking tens of minutes to analyse a single dataset^12,16^.

In this work, we present DH-PSF unmixing (DHPSFU), a simple and novel algorithm for analysing DH-PSF image data. Following detection of the individual 2D Gaussian-shaped peaks (i.e. separate detection of each of the two DH lobes) in the stack of raw images, DHPSFU identifies localisations through distance-based DH lobe pairing, which we demonstrate to be an accurate, sensitive and fast approach. The z-coordinate of each localisation is then determined using the rotation angle of the DH-PSF and a model obtained from fitting to calibration data. Additionally, DHPSFU extracts other characteristics of the localisation and applies filters based on specific criteria to enhance accuracy. We provide DHPSFU as a Fiji plugin with a user-friendly interface, making it accessible to users with no prior programming experience. The DHPSFU plugin not only identifies the 3D coordinates of DH-PSF localisations, but it also integrates other useful SMLM analysis tools such as PSF characterisation, temporal grouping of localisations, drift correction, and visualisation of the results in a convenient package. Our plugin is available on GitHub (https://github.com/TheLaueLab/DHPSFU_Fiji_Plugin), together with installation and usage instructions and datasets are available on Zenodo^21^.

## Results

### Principle of DHPSFU

**Fig. 1:**
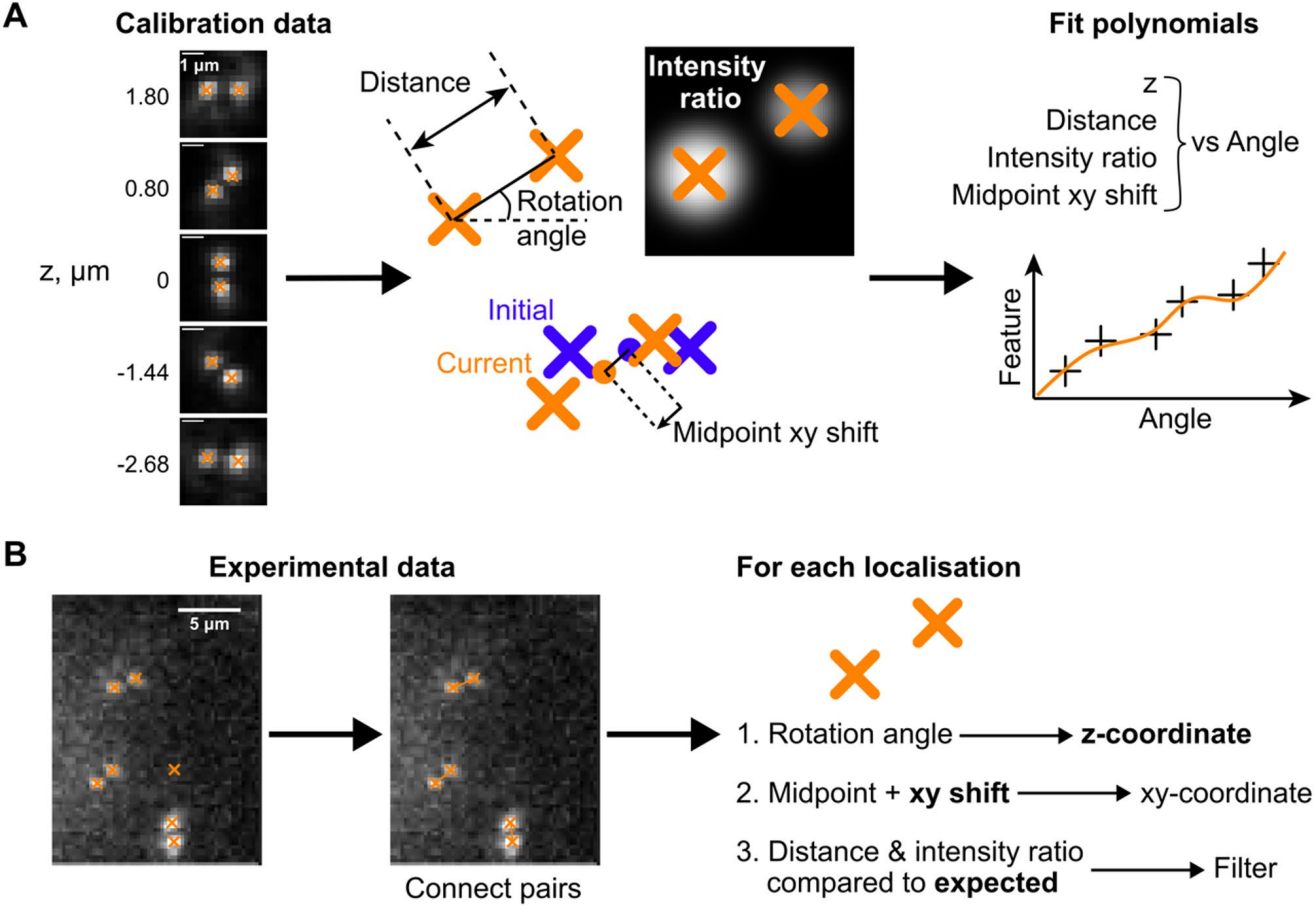
Working principle of DHPSFU. (**A**) Individual peaks in the calibration data are found using a 2D localisation algorithm (orange crosses). For each frame in the calibration dataset, pitch, distance between points, intensity ratio, and midpoint shift compared to the first frame in the stack are calculated. These features are linked to the known z-coordinate from the calibration experiment, and polynomial functions (orange line) are fitted to the data (black crosses). (**B**) In the experimental dataset, predetermined peaks are connected based on the expected pairwise distance, which excludes unpaired noise peaks. For each pair: (1) The z-coordinate is predicted based on the rotation angle; (2) xy-coordinates are determined from the midpoint and the PSF-induced shift is corrected for using the calibration; (3) localisations that have large deviations from the expected interlobe distance or lobe intensity ratio are filtered out. Scale bar, 5 μm.

Given a set of 2D coordinates of individual DH lobes and a calibration dataset, DHPSFU converts the former into 3D SMLM data through distance pairing and filtering. The algorithm operates in two stages: (1) development of a model of the DH-PSF based on calibration data, and (2) experimental data analysis. First, an experimental calibration z-stack is generated by imaging fluorescent beads on a coverslip and stepping through small axial steps of 10–100 nm. For each image, the lateral localisations of two lobes of a single DH-PSF are determined using an algorithm of choice (in our case GDSC Peakfit^22^) and based on these positions, the angle of the DH throughout the axial range is calculated (Fig. 1A). The dependencies of z-position, PSF midpoint position in x and y, interlobe distance and lobe intensity ratio on the angle are then fitted with polynomial functions, such that these features can be determined for experimental data. For the experimental data, the pairwise Euclidean distance matrix is calculated for all peaks, and the peaks likely originating from the same molecule are paired if the distance between them falls within the expected range (Fig. 1B). The rotational angle is then used to determine the z-coordinate of each molecule, and other features are used to filter for high-confidence localisations. The benefits of this approach compared to template matching are simplicity and processing speed.

### Benchmarking DHPSFU performance using simulations

To evaluate the capabilities of DHPSFU, we benchmarked its localisation detection performance with two commonly used software packages (EasyDHPSF and SMAP) by comparing three measures: (1) the number of true localisations detected divided by the total number of detected localisations (precision); (2) the number of true localisations detected divided by the total number of true localisations (sensitivity), and (3) the distance of the detected localisation to the true localisation (localisation accuracy). We assessed three different simulated datasets to establish performance over a range of microscope parameters: 1) The established super-resolution fight club^20^ reference standard (Sage et al. datasets: MT0.N1.LD and MT1.N1.LD); 2–3) Data simulated with a PSF determined^23^ from an experimental calibration using a (2) commercial (200 nm camera pixel size) and (3) bespoke 3D-printed^24^ (100 nm camera pixel size) DH-PSF mask. While the Sage et al. dataset acts as an excellent benchmark, it relies on interpolation in the z axis to generate the PSF, which could mask intensity fluctuations present in a real mask. By instead using phase retrieval^23^ we could generate PSFs that represent real microscopes without interpolation.

**Fig. 2:**
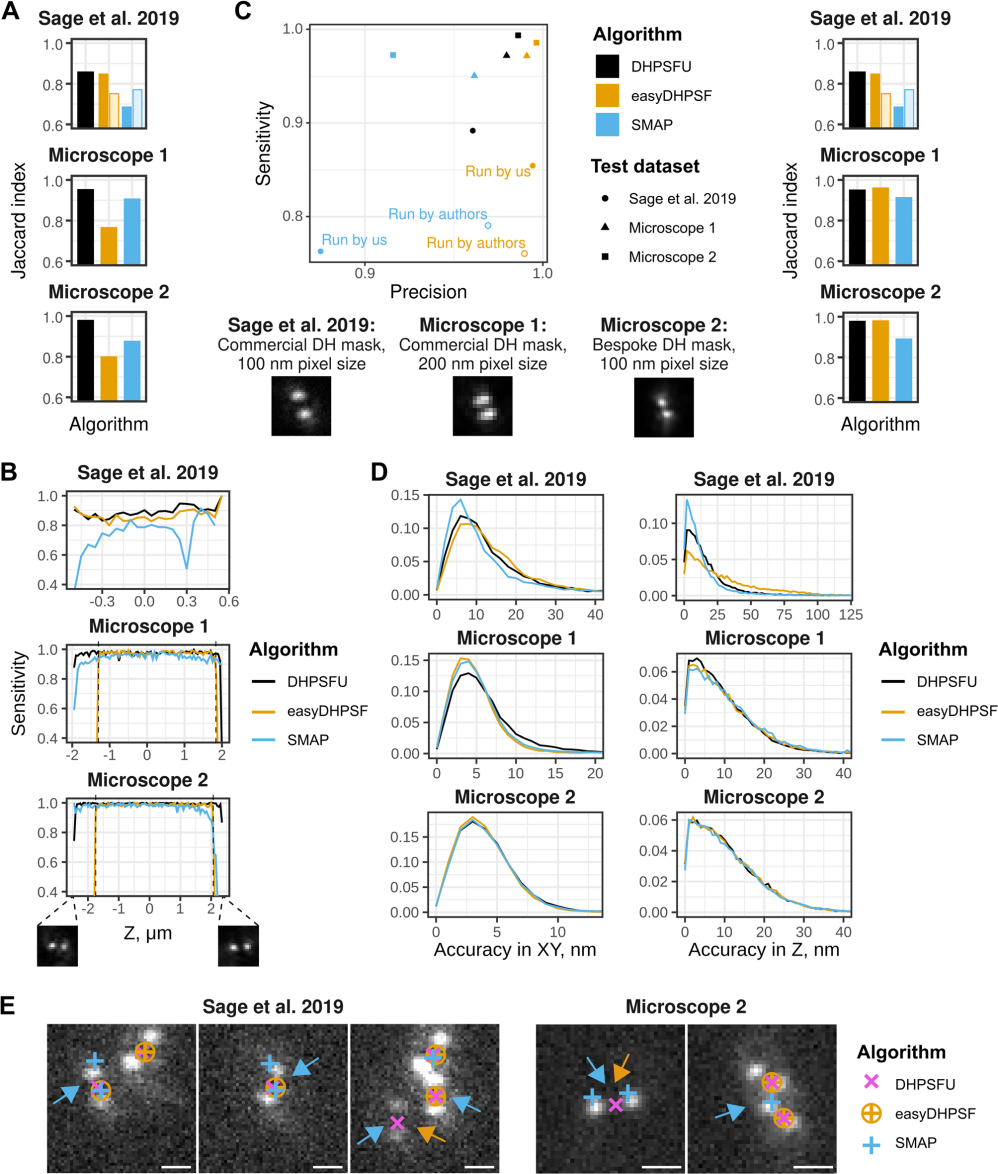
Benchmarking DHPSFU performance against established algorithms. **(A)** The Jaccard index (the number of true localisations divided by the total number of true plus false localisations detected) for each method. (**B)** The dependence of each method’s sensitivity on the z-coordinate of the localisation. The two images illustrate the near-horizontal orientation of the DH-PSF close to the extremes of the z-range in the ‘Microscope 2’ dataset. The dashed lines indicate the z-range to which the plots in C are restricted. **(C**) The performance of the algorithms in the “optimal” z-range. Left, a plot of sensitivity (the number of true localisations detected divided by the total number of true localisations) against precision (the number of detected true localisations divided by the total number of detected localisations) for the three processing methods. Right, the Jaccard index for each method. In A and C, the lighter points and bars represent the values taken from the Sage et al. dataset^20^, where the Sage et al. datasets were analysed by the method developers themselves. Bottom left, description and sample PSF close to z = 0 for the three microscopes used in this study. **(D)** The localisation accuracy in XY (left) and Z (right) for each microscope and algorithm. **(E)** Close-up views of regions from the Sage et al. datasets showing: *(1)* the splitting of a single localisations into two, merging of two localisations into one, or missed localisations by SMAP (blue arrows); and *(2)* the localisations missed by EasyDHPSF (orange arrows). Scale bars, 1 μm.

We first considered a combined measure, the Jaccard index (the number of true localisations divided by the total number of true plus false localisations detected). We found that DHPSFU (0.86–0.98) outperformed SMAP (0.69–0.91) and EasyDHPSF (0.76–0.85) under all conditions (Fig. 2A). This remained true when comparing the performance of DHPSFU (0.86) when processing the Sage et al. dataset^20^, to where the EasyDHPSF (0.75) and SMAP (0.77) algorithms were run by the authors themselves (Fig. 2A, hollow bars).

Unlike the Sage et al. dataset^20^, the two datasets we generated to test the three algorithms utilise nearly the full z-range of the DH-PSF, requiring distinction between similar rotation angles approaching 0 and 180 degrees. An ability to utilise the complete z-range is important when imaging thick samples such as mammalian cells, and we therefore analysed the sensitivity of the methods as a function of z (Fig. 2B). DHPSFU performed well throughout the z-range, while SMAP displayed mildly lower sensitivity and EasyDHPSF worked poorly at the extremes of the DH-PSF. While analysing the data with EasyDHPSF, we found that the thresholds for template matching of near-horizontal rotation angles had to be set to much higher values than for the rest of the PSF – lowering these thresholds led to a sharp increase in the number of false localisations. During our investigation we found that the interlobe distance limits are hard-coded in EasyDHPSF and this range is exceeded at the extremes of the PSF z-range in our simulations, which explains the sudden drop in sensitivity of EasyDHPSF.

To provide a fairer comparison, we further considered the localisations within a restricted z-range in which the PSF interlobe distance did not exceed the hard limits set by EasyDHPSF (3,196 nm for Microscope 1 and 3,840 nm for Microscope 2, indicated by dashed lines in Fig. 2B). We found that EasyDHPSF performed well in terms of sensitivity (0.80–0.98) and very well in terms of precision (> 0.99 for all datasets), whilst SMAP performed worse (Fig. 2C). DHPSFU demonstrated the highest sensitivity (0.90–0.99) while also achieving high precision (0.96–0.99; Fig. 2C). This analysis indicated that EasyDHPSF (Jaccard index of 0.85, 0.96 and 0.98 for the Sage et al. dataset, Microscope 1 and Microscope 2 respectively) and DHPSFU (Jaccard index of 0.86, 0.95 and 0.98) performed similarly well within the restricted z-range, while SMAP (Jaccard index of 0.69, 0.92 and 0.89) was inferior.

To assess how accurately the x, y and z coordinates of the localisations are determined by each algorithm, we calculated (for each localisation) the distance between the coordinates estimated by the software and the ground truth (localisation accuracy) and plotted the distribution of those distances. Generally, all three software packages achieved similar localisation accuracy in x, y and z, although the actual results varied according to the test dataset studied—likely due to the different signal-to-noise (S/N) ratios, pixel sizes and other parameters (Fig. 2D). The largest difference was found in z for the Sage et al. dataset^20^: the median distance between the true and predicted 3D-coordinates was 14.8 nm for SMAP, 18.9 nm for DHPSFU, and 28.8 nm for EasyDHPSF. However it should be noted, that this difference was not observed for our simulations (Microscope 1 and 2; Fig. 2D) and for the Sage et al. dataset, SMAP had much lower sensitivity (0.76 vs. 0.89 for DHPSFU), potentially indicating bias towards brighter localisations. Figure 2E shows representative examples where DHPSFU can identify localisations missed by EasyDHPSF or missed/overfitted by SMAP (Orange and blue arrows, Fig. 2E). Overall, DHPSFU achieved higher sensitivity largely due to its ability to work well with near-horizontal localisations. The main differences observed between Microscope 1 (commercial phase mask, 200 nm pixel size) and Microscope 2 (bespoke phase mask, 100 nm pixel size) was the overall higher Jaccard index (Fig. 2A-C) and improved localisation accuracy (Fig. 2D) for Microscope 2. We attribute this improvement to the higher NA (1.35 vs 1.27) and smaller pixel size of Microscope 2 as 100–150 nm is ideal^25^ in terms of matching the standard deviation of the PSF. However, it is worth noting that larger pixel sizes can be useful for low-photon localisations, as in the case of single-molecule tracking, which Microscope 1 was developed for^13^.

It is important to note that DHPSFU cannot correctly assign a z-coordinate to a localisation whose rotation angle is greater than 180 degrees. Such localisations are clearly observed in real image data, and although DHPSFU may be able to filter them out based on the distance between the lobes and their intensity ratio, that is not always possible. It is also crucial to verify that the PSFs in the calibration series do not cover more than 180 degrees. For these reasons we provide users with diagnostic plots at the end of the calibration procedure that can be used to define an appropriate angular range for model fitting. Any localisations in the experimental dataset whose pitch is beyond this range are then excluded by the software.

### Comparing the performance of algorithms on experimental data

Encouraged by the excellent performance of DHPSFU on simulated data, we investigated how the different algorithms performed when analysing experimental data. We used the resPAINT^16^ approach to acquire DH-PSF SMLM data of the plasma membrane of a Jurkat T cell. We used wheat germ agglutinin (WGA) labelled with HM-SIR that intermittently binds to glycosylated proteins on the cell surface. The spontaneous blinking of HM-SiR greatly increases the contrast in point accumulation for imaging in nanoscale topography (PAINT) experiments, which we applied to capture 75,000 frames of SMLM data with an exposure time of 20 ms.

**Fig. 3:**
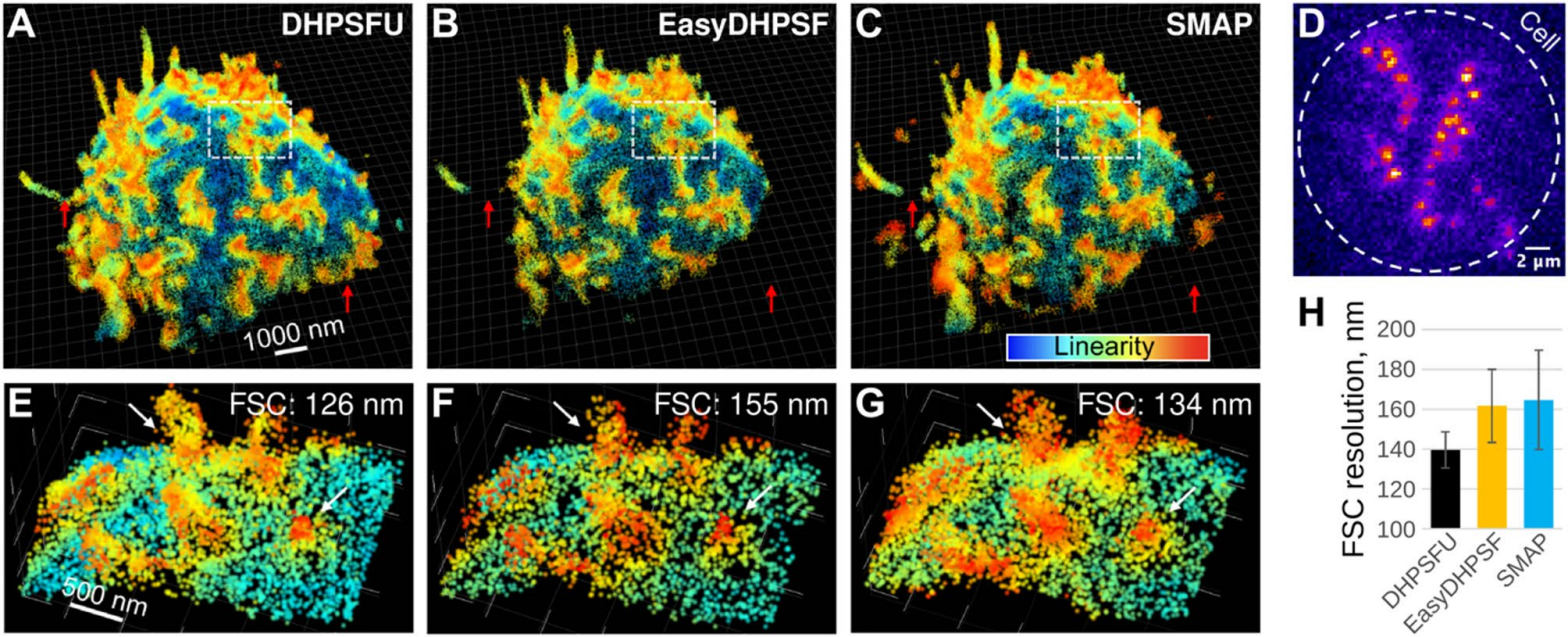
DHPSFU achieves the highest resolution for densely labelled experimental data. **(A-C)** resPAINT data showing the apical plasma membrane of a Jurkat T cell. Data was analysed using DHPSFU (**A**), EasyDHPSF (**B**) and SMAP (**C**). Data has been filtered by local density within 200 nm to remove noise. The colour represents linearity determined using principal component analysis, which helps identify cellular structures. The red arrows show areas where only DHPSFU captures membrane topography. **(D)** Representative raw resPAINT data, demonstrating high localisation density. The white circle shows the approximate outline of the cell. **(E**–**G)** Zoom-in of white boxes in **A–C**. The white arrows show areas where only DHPSFU provides a dense sharp representation of the membrane topography. The Fourier shell correlation (FSC) value is quoted for each condition. **(H)** Fourier shell correlation was used to assess the resolution of **A–C** (see main text).

Figures 3A–C show the result of analysing the experimental data using DHPSFU, EasyDHPSF and SMAP. The extended axial range of DHPSFU is immediately clear. Red arrows in Fig. 3A–C show areas where EasyDHPSF and SMAP miss large sections of the cell membrane that are captured by DHPSFU. The missing areas are mostly towards the bottom of the cell, suggesting a limited z range of both EasyDHPSF and SMAP. In the case of EasyDHPSF, this is likely due to the hard-coded interlobe distance limit. It is also clear that EasyDHPSF produces high-resolution but sparse data, whereas SMAP achieves high localisation density, but structures appear blurry. This is likely caused by the high-density labelling used in our experiment (Fig. 3D), which we chose to maximise collection speed and to demonstrate DHPSFU performance under challenging experimental conditions. These observations are further confirmed by considering the zoomed-in region in Fig. 3E–G. Again, only DHPSFU achieves high localisation density with well-defined structures. We quantified the resolution of the volumes in Fig. 3E-G using Fourier shell correlation (FSC^26^) analysis showing that DHPSFU achieves the best resolution (126 nm), when compared to EasyDHPSF (155 nm) and SMAP (134 nm). Finally, we applied FSC analysis to the entire dataset. To simplify analysis, we split the data into 1 × 1 × 1 μm voxels and only analysed voxels with > 1,000 localisations. Figure 3H shows the average FSC values for the three algorithms when only considering voxels where all three algorithms had > 1,000 localisations. DHPSFU clearly achieves the highest resolution (140 nm) between the algorithms (EasyDHPSF: 162 nm; SMAP: 165 nm), with a relative improvement of approximately 15%.

### DHPSFU outperforms other algorithms in challenging conditions

Having validated the performance of DHPSFU on high-quality, “easy” simulated datasets, as well as experimental data, we proceeded to compare the three algorithms under more challenging conditions by analysing simulated images with lower S/N or higher localisation densities. To provide a fair comparison, this benchmarking was done under the restricted z-range where EasyDHPSF shows adequate performance.

**Fig. 4:**
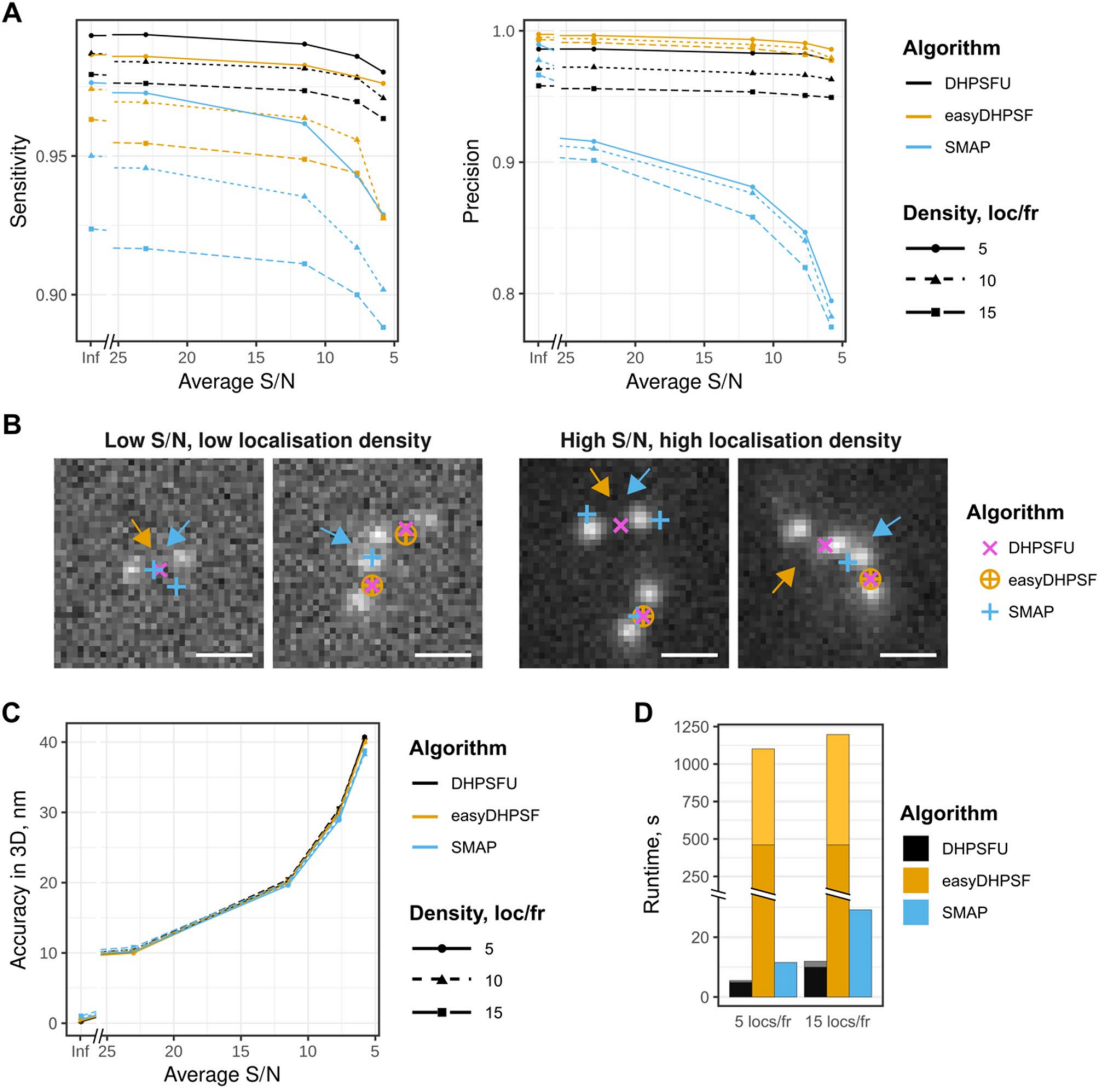
DHPSFU performance under challenging conditions. **(A)** The algorithms’ sensitivity and precision (defined as in Fig. 2) at different average signal-to-noise (S/N) ratios and localisation densities. **(B**) Examples of frames from a low-S/N dataset (left; S/*N* = 5.8, 5 localisations per frame) and a high-density dataset (right; S/*N* = 23, 15 localisations per frame), illustrating the splitting or merging of localisations by SMAP (blue arrows) and failure to detect near-horizontal PSFs by EasyDHPSF (orange arrows). Scale bars, 1 μm. **(C)** The 3D localisation coordinate precision reached by each algorithm in different conditions, defined as the median distance between each detected localisation and its true position. **(D)** The runtime of each algorithm for two datasets with intermediate S/N, 5 or 15 molecules per image frame on average, and 5,000 frames. The darker and lighter colours show the portion of time necessary for the first (GDSC PeakFit for DHPSFU, template matching for EasyDHPSF) or the second (DHPSFU algorithm itself, double-Gaussian fitting for EasyDHPSF) step of the method, respectively. The times taken for calibration are not included.

DHPSFU demonstrated the highest sensitivity, retrieving the highest number of localisations across all conditions while retaining high precision (Fig. 4A). Notably, while EasyDHPSF initially achieved a similar performance to DHPSFU, EasyDHPSF was much less stable with respect to noise and density. SMAP exhibited lower sensitivity (0.92) than DHPSFU (0.98), and its precision was more severely affected by decreasing S/N (Fig. 4A). At the lowest S/N and highest density, the three algorithms DHPSFU, EasyDHPSF and SMAP achieved a Jaccard index of 0.92, 0.91 and 0.71 respectively (Fig. 4A). Further investigation revealed that at low S/N, SMAP frequently misidentified single DH-PSFs as two distinct localisations, whereas at high localisation densities it tended to merge neighbouring localisations (see e.g. the localisations labelled with blue arrows in Fig. 4B). EasyDHPSF, on the other hand, failed to detect localisations with near-horizontal PSFs (see e.g. the localisations labelled with orange arrows in Fig. 4B). We also evaluated the accuracy of 3D coordinate determination for each method. Localisation coordinate accuracy was largely unaffected by localisation density, but was significantly influenced by the S/N and all three algorithms achieved similar results, suggesting optimised analysis (Fig. 4C).

### The DHPSFU algorithm is computationally efficient

Finally, we compared the processing times required to analyse two typical datasets of 5,000 frames, with lower or higher localisation density, using all three algorithms (Fig. 4D). In all cases, the time taken for calibration was excluded from the comparison, but for DHPSFU the time required for the initial detection of individual peaks using GDSC PeakFit^22^ was included. EasyDHPSF required approximately 20 min for analysis in both cases, whereas SMAP completed the task in 10–30 s. DHPSFU was the fastest, with runtimes of 4 and 11 s. This performance is very useful for rapid processing of images whilst acquiring data. It should be noted that SMAP offers GPU-acceleration, which was not employed here, our comparison is strictly CPU based. Overall, DHPSFU proves to be a highly efficient DH-PSF analysis algorithm, which for convenience of use we have implemented as a Fiji plugin (https://github.com/TheLaueLab/DHPSFU_Fiji_Plugin, available via ImageJ update sites). Matlab and Python scripts are also available together with usage instructions (https://github.com/TheLaueLab/DHPSFU) for adaptation and batch processing. A readme and user guide also explain how to access and use the Fiji Plugin, accessible through the Github link. To support reproducibility and adoption, we have provided sample datasets (simulation and experimental) as well as a sample analysis pipeline^21^.

## Discussion

We have shown that DHPSFU outperforms the popular algorithms EasyDHPSF and SMAP that are routinely used for DHPSF fitting in terms of precision, z-range and processing time. These, as well as most other algorithms, rely on some form of pattern matching such as template matching or cubic spline fitting, either during localisation detection or z-coordinate determination stages, or both. By taking the simple approach of coupling pairs of peaks using the distance matrix and confining the matching based on PSF-related features, we have demonstrated that we can improve both performance and processing speed. While we have achieved the highest Jaccard index (0.86) using an analytical approach for the low-density DH-PSF data from the Sage et al. dataset^20^, it should be noted that we are comparing our results, where we had access to ground truth, with author results that were achieved without ground truth knowledge. To provide a fair comparison we optimised EasyDHPSF and SMAP using the known ground truth, and we were able to improve the sensitivity of EasyDHPSF, but in no case could we outperform DHPSFU.

The superior performance also applied to the analysis of experimental data. Only DHPSFU was capable of capturing the full useful range of the DH-PSF (about 5 μm for our mask). While the performance of EasyDHPSF and DHPSFU were mostly similar in terms of precision and localisation precision for the simulated data, FSC analysis of the experimental data showed a substantial 16% improvement in resolution by DHPSFU. It should be noted that this comparison was done within the effective range of EasyDHPSF and that it therefore represents an unambiguous improvement by our algorithm. This improvement is likely related to the superior performance of DHSPFU under high density conditions, supported by the approximately 2x improvement in the total number of localisations. While SMAP detected a similar total number of localisations, cellular structures appeared blurrier under inspection and FSC analysis, suggesting poorer accuracy under these conditions.

Unlike EasyDHPSF and DHPSFU, SMAP is not specifically designed for DH-PSF microscopy. Instead, SMAP can be used to process images acquired with any type of PSF engineering. This is achieved by detecting localisations in a mean-filtered image, rather than the raw data. However, specifically for DH-PSF, this creates complications. The DH-PSF has an elongated shape with two lobes, and when acquired with typical experimental setups each of the lobes may have a long tail. Furthermore, the size of the DH-PSF may change as a function of z. Thus, it is often not possible to find a single optimal filtering radius applicable throughout the axial range of the PSF, and as a result, in some cases SMAP splits localisations into two and in others it merges nearby localisations (see e.g. the localisations labelled with blue arrows in Fig. 2D). Indeed, we noticed that for the Sage et al. dataset^20^, the dependence of SMAP sensitivity on z correlated inversely with the distance between the DH lobes (Fig. 2B). In contrast, DHPSFU’s simple localisation approach, which is adapted specifically for DH-PSF microscopy, circumvents these issues. Its stringency is further enhanced by the angle-dependent filtering of localisations based on the distance between the two lobes and their intensity ratio.

Deep-learning approaches to SMLM have seen major developments in recent years and methods such as DeepSTORM3D^27^ and DECODE^28^have started to outperform analytical approaches. In fact, DECODE achieves the highest Jaccard index for DH-PSF data from the Sage et al. dataset (0.98), which is much higher than our result (0.86). While it would have been valuable to compare the performance of DHPSFU with deep-learning approaches, this is a mammoth task worthy of its own study that is beyond the scope of this article. Some drawbacks of deep-learning methods in comparison to analytical approaches are worth mentioning. These algorithms require cumbersome training on simulated data that is representative of what is being imaged. Such training requires significant time (10s of hours to train a model), expertise in deep learning, specialist high-performance computers and a deep understanding of the PSF of the microscope. Beyond this, the algorithms are known to hallucinate data, particularly under challenging or unknown conditions^29,30^. Such hallucinations are observed in imaging experiments and they mostly stem from insufficient or unrepresentative training data^31^, which can be avoided through careful experiment/analysis design and experimental benchmarking as done for DECODE^28^. There are also issues related to variations in PSF over time due to temperature changes or optical component drift as well as the presence of aberrations induced by refractive index mismatch due to the cell^32^. These aberrations are particularly problematic when performing large DOF imaging using DHPSF far above the coverslip. The ability of a typical SMLM user to implement and benchmark these approaches effectively is questionable in the current state of deep-learning approaches for SMLM.

Despite the challenges present, it is worthwhile mentioning efforts by others to streamline the deep learning for SMLM process and improve its user friendliness. In-situ^33^ and field-dependant^32^modelling reduce effects of aberrations. Cloud-based approaches are being developed to remove the requirement of specialist equipment and to streamline the training/analysis process^34^. Transfer-learning can be used to quickly retrain base models using a new daily calibration^35^. There are also methods that apply deep-learning approaches onto the localised data that affords fast processing times^36^. Ultimately, we envisage two types of users: (1) An expert with a dedicated team trying to maximise performance under low photon conditions or dense datasets for high throughput or live cell imaging and (2) a microscopist or biologist who has acquired some DH-PSF data and would like to analyse it in a simple and effective manner. Our algorithm has been designed with use case 2 in mind, and while DHPSFU seems to outperform similar algorithms, we would always recommend alternative approaches such as deep learning for use case 1, which is what they were designed for^27,28^. There are also analytical methods such as 3B^37^ and HAWK^38^ that can improve localisation performance under dense conditions, but our goal in this work was to provide a simple and effective direct analytical DHPSF localisation approach, which we have achieved.

While our algorithm is the fastest among those studied in this work, there are faster methods, most of which rely on GPU-acceleration for localisation. Cubic spline approaches^19,39,40^ (including SMAP) are highly compatible with the GPU architecture and can achieve > 10^5^ localisations/s (compared to 6,000/s for DHPSFU). However, this is also the case for Gaussian fitting^41^ and DHPSFU itself could be adapted to GPU-acceleration, which we plan to implement in the future as both Euclidean distance matrix calculations and matching are GPU compatible.

The creation of simulated data for benchmarking based on experimental data can be challenging in terms of precise definition of the ground truth. For the super-resolution fight club, the authors defined the ground truth as the centre of mass at z = 0. This can be problematic for the DH-PSF as the intensity ratio between the lobes tends to vary due to camera noise, rolling shutter effects and other external influences. Here, we instead relied on the mid-point of the two separately identified lobes. While this is still influenced by noise, the influence is minimised as the fits are concentrated on the lobes.

A second problem in benchmarking relates to how to model the PSF. For the Sage et al. simulation, interpolation in z was employed to generate data at any 3D position. Such an approach works well if the z steps are very small, but acquiring such a calibration stack across a large z-range is often associated with significant stage drift. We instead opted for phase retrieval^23^, which is compatible with much larger step sizes and thus faster calibration acquisition and less drift, to achieve a realistic PSF that still relies on experimental data. An additional benefit of phase retrieval is that a PSF can be generated at any 3D position with a known ground truth without relying on lateral shifts that require additional interpolation.

Finally, to account for the variation in PSF features on the z-coordinate, a function must be fitted to the calibration data. Polynomial or spline functions are typically used for this purpose. For high-quality data and a well-conditioned PSF, spline (as used in EasyDHPSF) is superior as it can take small local variations into account. However, overfitting can become a problem when either the calibration stack or the data are noisy. In this case, we found that a 10-15-degree polynomial fit was more stable and reliable, which is what we have used in this study. However, the DHPSFU plugin offers both options.

## Conclusion

We developed a simple algorithm, DHPSFU, for 3D DH-PSF localisation analysis that relies on accurately pairing DH lobes using a distance matrix. By benchmarking DHPSFU performance on an external dataset as well as our own simulations based on experimental data, we demonstrate that our approach outperforms the commonly used dedicated algorithm EasyDHPSF and the general cubic spline method SMAP, in particular for densely labelled noisy data. The simplicity of the approach also leads to improved CPU processing times (DHPSFU: 11 s; SMAP: 30 s; EasyDHPSF: 20 min) and we provide a Fiji plugin along with Matlab and Python scripts for user friendliness and customisation. Overall, DHPSFU offers fast, accurate and simple analysis of DH-PSF data with unrivalled performance among analytical 3D localisation algorithms.

## Methods

### Overview of the Fiji plugin

To enhance the accessibility of DHPSFU for users with limited knowledge of computational analysis, an ImageJ plugin incorporating the DHPSFU algorithm was developed. This plugin provides a comprehensive suite of tools for analysing 3D SMLM and Single-Particle Tracking (SPT) data obtained using DH-PSF microscopy, offering a user-friendly interface and fast performance with the following key functionalities:

#### DHPSFU

This function converts a list of 2D localisations into 3D coordinates using the DHPSFU algorithm with filters to remove ambiguous localisations. Diagnostic plots are displayed at the end of the procedure to help the user verify that the calibration has been performed successfully. The resulting 3D coordinates can be directly visualised using ViSP^42^.

#### Drift correction

The Drift Correction function mitigates lateral drift during image acquisition by employing an image cross-correlation algorithm^43^.

#### Blinking correction

The Blinking Correction function groups 2D/3D localisations that occur in the same place and in consecutive frames to correct for blinking fluorophores that are visible for several frames. This function can also be used for 2D/3D single molecule tracking.

#### Overlay

The Overlay function superimposes localisations from the results dataset onto the image, updating dynamically as the user switches between image frames. This helps visualise which localisations have been accurately identified or missed by DHPSFU.

#### Load file localisations

The Load File Localisations function loads lists of 2D/3D localisations from files into Fiji memory.

#### View and save localisations

The View and Save Localisations function allows the conversion of localisation results stored in the ImageJ memory into different formats, such as tables, images, and files. Users can change the distance unit and modify the data directly through an interactive table.

DHPSFU per se does not require the raw images, since the algorithm works on the lists of pre-fitted peaks stored in Fiji memory. To enable performing the full analysis cycle from raw images to 3D localisations, the DHPSFU plugin requires the GDSC Peakfit library as a dependency, and some of its useful functions can be applied to DHPSFU results. The software is distributed through an ImageJ update site, which hosts all the necessary files for the plugin installation. Additionally, guidance for use of the key functions is available when clicking the help button in the graphical user interface (GUI). The plugin also supports ImageJ macros for batch processing. Collectively, these features make the DHPSFU plugin a comprehensive tool for advanced imaging analysis, thereby facilitating its widespread adoption and effective utilisation within the DH-PSF imaging community.

### Calibration

The calibration data for DH-PSF microscopy (and PSF-engineering approaches in general) typically consists of a z-stack of images of point sources (usually a sample of fluorescent beads on a coverslip) with a small (10–100 nm) step size. For calibration, DHPSFU specifically requires as input a vector of coordinates of the two individual peaks in each image, representing the two DH lobes of a particular fluorescent bead as it is imaged throughout the desired axial range. For each image, the algorithm calculates the pitch of the double helix (rotation angle), and then a 15-degree polynomial function is fitted to these values to relate the rotation angle to the z-coordinate (Fig. 1A). In real optical systems, imperfections in the shape of the 3D PSF can cause the centre of the PSF to shift in the x-y plane as a function of z (also known as wobble^20^). To account for this, DHPSFU also determines the shift of the midpoint between the two DH lobes for each image (in comparison to the first reference frame in the stack). In addition, the distance between the two lobes and their intensity ratio are measured. Polynomial models are then fitted to each of these sets of data, and these models are used in the experimental data analysis (Fig. 1A). Of note, it is important to limit the range of the calibration series to 180 degrees to avoid ambiguities in the angle to z-coordinate correspondence, which would otherwise lead to erroneous function fitting. To facilitate this, DHPSFU produces diagnostic plots for the calibration, and an option to exclude the extreme ends of the calibration series is provided.

### Experimental data analysis

For each image in the experimental dataset, peaks likely originating from the same molecule are identified by calculating the pairwise Euclidean distance matrix. Distances that fall within the user-defined expected range are paired together. The rotation angle is then calculated for these pairs and used to determine the z-coordinate. The x, y-coordinates of the molecule are determined by calculating the midpoint between the DH lobes, which is then corrected by the expected midpoint shift at that z-coordinate. In addition to the midpoint shift, the expected DH lobe separation and intensity ratio of the PSF at that z-position are also computed. Localisations that deviate too far from the expected values based on the calibration data are discarded using filters with user-specified tolerance ranges relative to the calibration. Finally, localisations with a rotation angle beyond the extremes of the calibration series are removed, since their z-coordinate cannot be identified reliably.

### Simulation of DHPSF image stacks

To evaluate the performance of the DHPSFU algorithm in various imaging conditions, two simulated test datasets were generated, and one was obtained from previous published work.

#### Simulated dataset 1 (Fig. 5)

The first dataset was generated from real calibration series data recorded using a custom-built DH-PSF microscope^13^. Specifically, images of single fluorescent microspheres (TetraSpeck™ Microspheres, 0.1 μm, fluorescent blue/green/orange/dark red, ThermoFisher, T7279) were acquired at a series of z-positions (119 positions with an axial step of 33.3 nm) spanning the full range of the DH-PSF (4 μm), enabling the actual x-, y-, and z-coordinates of the microspheres to be recorded. To get an estimate of the overall PSF, five beads from the calibration were chosen and fused together to form an average PSF using the script from Sage et al.^20^The pixel size of the calibration data was 210 nm, which the fusion script upscaled to 100 nm using xy interpolation. To generate a complete 3D DH-PSF without z-interpolation, we used the vectorial implementation of phase retrieval (VIPR)^23^, which determines the phase mask that best represents the experimental data. The result was a realistic phase mask that captures the experimental data and can generate a PSF at any xyz.

This simulated 3D DH-PSF model was then used to produce a calibration stack that matched the experimental axial step size. A separate stack was generated consisting of 500 frames of DH-PSF at varying x, y and z positions. The x and y positions were varied with subpixel precision. These simulated DH-PSF images were then randomly distributed across 5,000 blank frames, with multiple DH-PSFs appearing in each frame to simulate fluorophores at different axial positions within a typical field of view. To replicate realistic intensity fluctuations observed in actual imaging, the intensity of each processed DH-PSF image was randomly varied by a multiplicative factor ranging from 1 to 1.2. Simulations with varying Gaussian-distributed background noise and different numbers of DH-PSF per frame (5, 10, or 15) were generated to assess the performance of the DHPSFU algorithm across different signal-to-noise ratios (S/N) and localisation densities. The resultant stacks were then normalised to have the same mean background levels (50), but with varying intensities implemented by dividing all pixel intensities with a corresponding factor. The approximate S/N values in plots were defined as mean brightest pixel value across localisations divided by the standard deviation of the Gaussian-distributed noise.

The ground truth of the simulated datasets was defined as the sum of the subpixel xy position and the coordinates where the simulated DH-PSF was pasted within the blank frame. The ground truth z-coordinates were extracted from the original z-position in the *fusion stack.*

#### Simulation 2

A second dataset was generated following the same procedures as the first, except that the calibration data was obtained from a different DH-PSF microscope^24^ equipped with a different objective (1.35 NA silicone oil instead of 1.27 NA water), phase mask (3D printed instead of commercial), camera (sCMOS instead of EMCCD), axial step size (60 nm), and pixel size (130 nm). This additional dataset allowed us to evaluate the robustness and consistency of DHPSFU across varying optical setups and imaging conditions.

#### Simulation 3

The third (Sage et al.) dataset was sourced from an open-source competition^20^, established to evaluate the performance of SMLM localisation extraction software. This published dataset was selected because it has previously been used in the field, providing a solid benchmark for comparison, and it also enabled a direct comparison with published results.

**Fig. 5:**
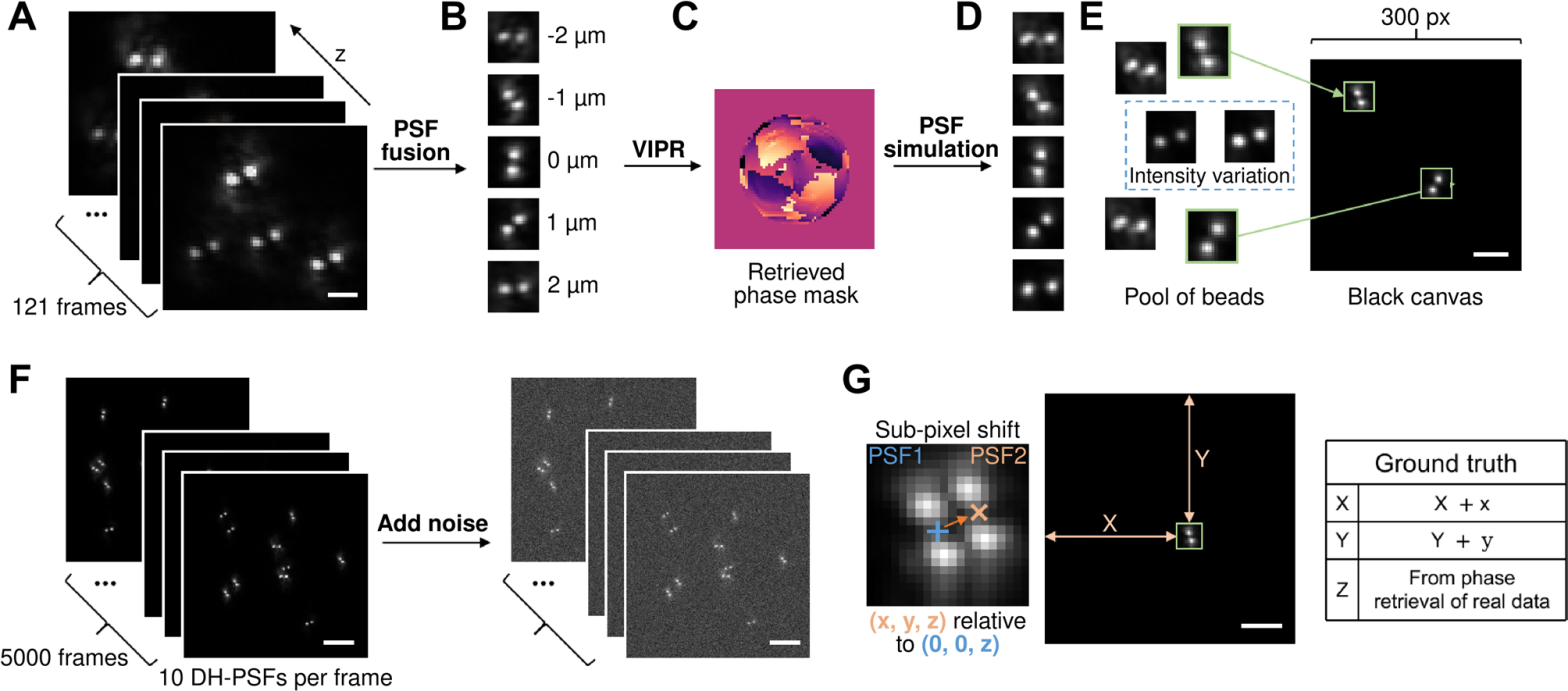
Procedure for simulating DH-PSF test datasets. (**A**) A typical experimental data series comprising frames of fluorescent nanoparticles acquired at different axial positions in the form of DH-PSFs. (**B**) Beads were fused together to form an average PSF. (**C**) Vectorial implementation of phase retrieval (VIPR) was performed on the fused PSF to model the phase mask that best describes the experimental DH-PSF. (**D**) With the retrieved phase mask, a stack of DH-PSFs ranging from − 2 μm to + 2 μm was simulated. (**E**) A pool of beads was then created for the generation of simulated data. Intensity variations, as indicated by the rectangular selection, were introduced to these images, and the modified DH-PSFs were randomly distributed across an empty canvas. (**F**) 5,000 simulated image frames were generated for each test stack with each frame containing ten DH-PSFs. Subsequently, Gaussian noise was introduced to the image stack to mimic real-world images. (**G**) Ground truth determination. The simulation code enables a DH-PSF to be generated at any position PSF2 (x, y, z) relative to the origin position PSF1 (0, 0, z), where (x, y) is a random subpixel shift. During the simulation process, the pixel-level position where the bead image was placed, (X, Y), is recorded. The ground truth coordinates of the bead is the sum of these two positions. The Z-coordinate is determined and recorded during the phase retrieved process. Scale bars, 2 μm (**A**, **B**) and 5 μm (**D–F**).

### Analysis comparison for different DHPSF algorithms

To evaluate the accuracy and robustness of the DHPSFU algorithm, three different simulated datasets with known x-, y-, and z-coordinates were used as ground truth benchmarks^20^. These test datasets were then analysed with DHPSFU, as well as with EasyDHPSF (v2.1, https://sourceforge.net/projects/easy-dhpsf/) and SMAP (Superresolution microscopy analysis platform 2020, https://github.com/jries/SMAP) for comparison. These two algorithms were chosen since they employ very different approaches to DH-PSF localisation detection and coordinate determination. EasyDHPSF utilises template matching to recognise candidate peaks, followed by 2D Gaussian fitting to determine the axial coordinate of each molecule. In contrast, SMAP identifies localisations in the x, y plane by peak finding in a mean-filtered image and determines the z-coordinate by fitting to a cubic spline model of the PSF constructed from the calibration data. Moreover, in a 2019 evaluation^20^, SMAP was the best-performing software in the DH-PSF category. In each case, the data were analysed following the authors’ guidelines, and the same bead(s) were used for calibration of all three algorithms. Parameters were then further adjusted empirically, guided by visual inspection of the overlay of detected localisations on the raw data. Additionally, the results using a range of key parameters were also compared.

*DHPSFU.* We first employed the GDSC Peak Fit algorithm to locate the two individual DH-PSF lobes at subpixel resolution. We applied stringent parameter settings for the calibration data but used more relaxed thresholds for the experimental data to maximise the detection rate (Table S1). The list of peaks was then analysed with DHPSFU (Table S2).

*EasyDHPSF.* After calibration and template matching, the thresholds for the final fitting step were manually selected after inspecting the images of potential template matches. We noticed that this procedure is time-consuming and highly subjective – it also becomes less reliable at higher localisation densities, significantly impacting the results. The parameters used for EasyDHPSF processing are summarised in Tables S3, S5 and S6.

*SMAP.* In SMAP, the main user-defined parameters are the size of the mean filter and the threshold for picking localisations, both of which influence localisation detection but not 3D fitting. These two parameters were optimised together (Tables S4, S5 and S6).

For the Sage et al. MT0.N1.LD dataset^20^, following the original authors’ procedure, localisations near the borders were excluded from the analysis. In addition, the dimmest 25% of localisations were not considered, as they were not expected to be detected by the software. Finally, to account for potential uniform translation of all localisations, the lists of localisations were post-processed by adjusting the coordinates of all localisations by the mean difference between the true and detected positions of each localisation (excluding false-negatives and false-positives). This procedure was not necessary for the Microscope 1 and 2 simulations, since we tested and accounted for such translation by refitting the calibration stack.

### Algorithm runtime comparison

The runtime comparisons were performed using two 5,000-frame datasets with 5 or 15 localisations per frame and with an S/N of 6.6, on a PC with an Intel Core i7-1165G7 processor (4/8 cores, 2.80 GHz) and 32 GB RAM. The times were reported by the functionality built into each software package.

### Acquisition of DH-PSF calibration series

#### Microscope 1

The calibration series was acquired using a custom-built system in Epi illumination mode with a 638 nm diode laser combiner (180 mW 06-MLD-638, Cobolt, and 180 mW LBX-638-180, Oxxius). The illumination was focused onto the sample using a 60X water immersion objective (CFI Plan Apo IR 60XC WI 1.27 NA, MRD07650, Nikon). The emitted fluorescence was collected through a detection arm consisting of a 4f system with two 200-mm tube lenses (TTL200-A, Thorlabs) and emission filters by an EMCCD camera (Evolve 512, Photometrics). For DH-PSF imaging, a double-helix phase mask (Double Helix Optics) specific to the far red emission was integrated into the Fourier plane of the 4f system. The imaging pixel size of this system was measured to be 210 nm by a line grating target (R1L3S6P, Thorlabs).

Micro-Manager 2.0 was used to control the shutters, the sample stage, the integrated perfect-focus system (PFS) in the microscope body, and cameras, as well as to acquire images. Home-written Micro-Manager scripts were developed to acquire the calibration series.

#### Microscope 2

The calibration series was acquired using a custom-built system in Epi illumination mode with a 638 nm fiber-coupled laser (72 mW, LaserTree). The illumination was focused onto the sample using a 100X Silicone Oil immersion objective (CFI SR HP Plan Apo Lambda S 100XC Sil 1.35 NA, MRD73950, Nikon). The emitted fluorescence was collected through a detection arm consisting of a 4f system with two 200-mm tube lenses (TTL200-A, Thorlabs) and emission filters by a sCMOS camera (BSI Express, Photometrics). For DH-PSF imaging, a custom double-helix phase mask created using additive manufacturing^24^ was provided by Yoav Schechtman and integrated into the Fourier plane of the 4f system. The imaging pixel size of this system under 2 × 2 binning mode was measured to be 130 nm by a line grating target (R1L3S6P, Thorlabs).

Micro-Manager 1.4 was used to control the z-piezo stage (P72.Z100, CoreMorrow) and the camera, as well as to acquire images. Custom Micro-Manager scripts were developed to acquire the calibration series.

### Fourier shell correlation

Fourier shell correlation was used to assess the resolution of the experimental data analysed using the different algorithms. We used the CellSTORM FSC implementation^26,44^. First, the localisation data was randomly split into two sets and assigned to 5 × 5 × 5 nm voxels. A 3D Gaussian filter (3 × 3 × 5 pixels, corresponding to the expected 3D localisation precision) was then applied to the volumes to create representative images. These images were then passed to the FSC script and a 1/7 threshold was used to determine the resolution.

For the entire cell FSC estimation, this approach was applied to subsets of the 3D images. The complete cell data was split into 1 × 1 × 1 μm voxels. For any voxel that had > 1,000 localisations, the FSC was determined to estimate resolution. As there were different localisations for the different algorithms, we only considered voxels for which all three algorithms had > 1,000 localisations when performing the final FSC comparison.

### resPAINT cell membrane imaging

resPAINT was performed with WGA-HM-SiR labelling the cell membrane of Jurkat T cells on a custom-built DH-PSF microscope as previously described^16^. In short, fixed (0.8% paraformaldehyde and 0.5% glutaraldehyde, 20 min RT) Jurkat T cells were attached to a coverslip coated with Poly-L-lysine. The suspension was then replaced with 2% low melting point agarose containing fiducial markers (0.2 μm FluoSpheres Dark Red), that was used for drift correction. The sample was excited with a 640 nm laser using a 60 × 1.27 NA water immersion objective lens with a power density of 2 kW/cm^2^. The filtered fluorescence was collected on a Photometrics 512 Delta EM-CCD with a magnified pixel size of 207 nm and an exposure time of 20 ms.

The raw data, as well as the images of the fiducial bead, were then processed using the three algorithms with the parameters listed in Tables S1-S4. The position of the fiducial in the corresponding frame was then subtracted from each localisation’s coordinate to correct for drift during acquisition. Next, we corrected for multiple appearances of the same molecule in neighbouring frames by averaging the coordinates of all localisations separated by < 150 nm in xy and < 50 frames, using a custom script (https://github.com/TheLaueLab/trajectory-analysis). The super-resolution images were finally visualised using ViSP^42^.

## Supplementary Information

Below is the link to the electronic supplementary material.

Supplementary Material 1

## Data Availability

The experimental data and the simulation results that support the findings of this study are available in Zenodo with the identifier https://zenodo.org/records/16357203.
