# Supplementary material for: DHPSFU: a Fiji plugin for fast and accurate double helix-PSF 3D single-molecule localisation microscopy
Source: Sci Rep. 2025 Aug 20;15:30551. doi: 10.1038/s41598-025-15623-3 (PMC12368115; doi:10.1038/s41598-025-15623-3)
Supplement: Supplementary file 1 — Supplementary Material 1 [file 41598_2025_15623_MOESM1_ESM.pdf]

DHPSFU: A FIJI PLUG-IN FOR FAST AND ACCURATE DOUBLE HELIX-PSF 3D SINGLE-MOLECULE LOCALISATION MICROSCOPY: SUPPLEMENTAL DOCUMENT

Tables S1-S6 describe the parameters used to simulate and analyse the data used to create the figures in the manuscript.

Table S1: GDSC PeakFit parameters for the fitting SMLM data in Figure 2, 3 and 4. All other parameters were kept at default values. (*) Values for noise 0 dataset; for higher-noise datasets, these values were divided by a factor of (noise/50). This normalisation (see methods for description) was required for compatibility with EasyDHPSF and SMAP.

Parameters	Microscope 1	Microscope 2	Sage et al 2019	Experimental
Camera type	CCD	CCD	EMCCD	EMCCD
Camera bias	0.1* (1)	0.1* (1)	400	1
Camera gain	0.2* (2)	0.2* (2)	35	2
Camera read noise	0.2* (2)	0.2* (2)	7.1622	2
PSF	Circular Gaussian 2D	Circular Gaussian 2D	Circular Gaussian 2D	Circular Gaussian 2D
PSF parameter 1 (s)	1	2.19	2	1.3
Spot filter type	Difference	Difference	Difference	Difference
Smoothing2	1.2	1.0	2.0	1.5
Smoothing	0.58	0.4	0	0.7
Search width	0.78	0.98	0.9	0.98
Border width	1.14	1.16	0.88	1.16
Fitting width	4.04	3.64	2.14	2.74
Fit solver	LVM LSE	LVM LSE	LVM LSE	LVM LSE
Fail limit	3	3	3	3
Pass rate	0.5	0.5	0.5	0.5
Neighbour height	0.58	0.6	0.3	0.66
Residuals threshold	1.0	1.0	1.0	1.0
Duplicate distance	0.96	0.96	0.5	0.88
Shift factor	1.14	0.96	1.2	1.28
Signal strength	1	1	20	1
Min photons	1	1	150	1
Min & max width	0.44 & 1.7	0.44 & 2.0	0.4 & 1.7	0.52 & 2.0
Precision	2-20 nm	2-30 nm	50 nm	30 nm

Table S2: DHPSFU parameters for fitting the SMLM data in Figure 2, 3 and 4.

Parameters	Microscope 1	Microscope 2	Sage et al 2019	Experimental
Pixel size (nm)	200	100	100	207
Precision threshold (nm)	30	30	30	30
Calibration step size (nm)	33.3	60	10	40
Range to fit (frames)	(1, 120)	(1, 82)	(1, 151)	(1, 127)
Initial distance filter (pixel)	(3, 8)	(4.5, 10)	(7, 13)	(4.5, 8)
Max distance deviation	0.2	0.2	0.2	0.6
Max intensity deviation	1.5	1.5	1.5	0.6

Table S3: EasyDHPSF parameters for the SMLM data in Figure 2 and 3.

Parameters	Microscope 1	Microscope 2	Sage et al 2019	Experimental
Pixel size (nm)	200	100	100	207
Conversion gain	6.5	6.5	6	6.5
EM gain	250	250	300	250
Thresholds	287 233 242 239 252 211	82 102 103 100 97 102	50 50 50 50 50 50	190 190 225 230 230 250<br>100 100 100 100 100 200 (fiducial)

Table S4: SMAP parameters for the SMLM data in Figure 2 and 3.

Parameters	Microscope 1	Microscope 2	Sage et al 2019	Experimental
Calibration filter (pixel)	3	3	17	3
Calibration min distance (pixel)	10	10	25	10
Calibration ROI (pixel)	15	17	27	15
Calibration z-smoothing	0.1	0.1	0.1	0.1
Camera offset (ADU)	400	0	100	400
Conversion	0.026	0.026	0.15	0.026
Pixel size (nm)	200	100	100	207
Analysis ROI (px)	11	15	19	11
Analysis filter (px)	3.5	4.1	5.7	3<br>(5 for fiducial)
Analysis cutoff (photons)	1.5	0.4	8	6<br>(10 for fiducial)

Table S5: Common EasyDHPSF and SMAP parameters for the SMLM data in Figure 4.

Algorithm	Parameters	Value
**EasyDHPSF**	Pixel size (nm)	100
	Conversion gain	6.5
	EM gain	250
**SMAP**	Calibration filter (pixel)	3
	Calibration min distance (pixel)	10
	Calibration ROI (pixel)	17
	Calibration z-smoothing	0.1
	Camera offset (ADU)	0
	Conversion	0.026
	Pixel size (nm)	100
	Analysis ROI (pixel)	15

Table S6: Varied EasyDHPSF and SMAP parameters used for simulation datasets in Figure 4.

Dataset name	EasyDHPSF	SMAP	
	**Thresholds**	**Analysis filter (Pixel)**	**Cutoff (photons)**
Dens5_noise0	89 125 117 115 120 125	4.5	30
Dens5_noise5000	82 102 103 100 97 102	4.1	0.4
Dens5_noise10000	67 78 71 74 72 79	4.1	0.2
Dens5_noise15000	53 60 62 58 59 55	4.1	0.135
Dens5_noise20000	45 56 49 50 46 47	4.1	0.1
Dens10_noise0	71 88 82 79 84 88	4.5	30
Dens10_noise5000	62 78 72 74 74 80	4.1	0.4
Dens10_noise10000	52 67 58 62 61 67	4.1	0.2
Dens10_noise15000	45 53 52 50 50 55	4.1	0.135
Dens10_noise20000	37 44 45 41 42 50	4.1	0.095
Dens15_noise0	60 69 62 63 67 71	4.5	30
Dens15_noise5000	53 64 61 60 60 69	4.1	0.4
Dens15_noise10000	47 55 50 54 51 57	4.1	0.19
Dens15_noise15000	42 46 46 42 45 48	4.1	0.12
Dens15_noise20000	35 41 39 39 40 43	4.1	0.09
